# Impact of use of optical surface imaging on initial patient setup for stereotactic body radiotherapy treatments

**DOI:** 10.1002/acm2.12779

**Published:** 2019-12-13

**Authors:** Brian Leong, Laura Padilla

**Affiliations:** ^1^ Department of Medical Physics Memorial Sloan Kettering Cancer Center New York City NY USA; ^2^ Department of Radiation Oncology Virginia Commonwealth University Richmond VA USA

**Keywords:** initial positioning, interfraction variations, patient setup, SBRT, SGRT, surface imaging

## Abstract

**Purpose:**

To evaluate the effectiveness of surface image guidance (SG) for pre‐imaging setup of stereotactic body radiotherapy (SBRT) patients, and to investigate the impact of SG reference surface selection on this process.

**Methods and materials:**

284 SBRT fractions (SG‐SBRT = 113, non‐SG‐SBRT = 171) were retrospectively evaluated. Differences between initial (pre‐imaging) and treatment couch positions were extracted from the record‐and‐verify system and compared for the two groups. Rotational setup discrepancies were also computed. The utility of orthogonal kVs in reducing CBCT shifts in the SG‐SBRT/non‐SG‐SBRT groups was also calculated. Additionally, the number of CBCTs acquired for setup was recorded and the average for each cohort was compared. These data served to evaluate the effectiveness of surface imaging in pre‐imaging patient positioning and its potential impact on the necessity of including orthogonal kVs for setup. Since reference surface selection can affect SG setup, daily surface reproducibility was estimated by comparing camera‐acquired surface references (VRT surface) at each fraction to the external surface of the planning CT (DICOM surface) and to the VRT surface from the previous fraction.

**Results:**

The reduction in all initial‐to‐treatment translation/rotation differences when using SG‐SBRT was statistically significant (Rank‐Sum test, α = 0.05). Orthogonal kV imaging kept CBCT shifts below reimaging thresholds in 19%/51% of fractions for SG‐SBRT/non‐SG‐SBRT cohorts. Differences in average number of CBCTs acquired were not statistically significant. The reference surface study found no statistically significant differences between the use of DICOM or VRT surfaces.

**Conclusions:**

SG‐SBRT improved pre‐imaging treatment setup compared to in‐room laser localization alone. It decreased the necessity of orthogonal kV imaging prior to CBCT but did not affect the average number of CBCTs acquired for setup. The selection of reference surface did not have a significant impact on initial patient positioning.

## 1 Introduction

Optical surface imaging is an increasingly popular imaging modality used in radiotherapy for patient setup and monitoring. It provides real‐time feedback of the patient’s position with respect to a reference surface dictated by either the external body contour of the treatment planning CT, or a surface capture acquired with the surface imaging system cameras. At the time of treatment, the patient’s surface in the room is read by an optical system and automatically registered to the reference surface to calculate the deviation between the real‐time and expected treatment positions using six degrees of freedom (6DOF). This information can then be used to evaluate and readjust the patient’s setup from within the room without the use of ionizing radiation. More detailed descriptions of existing surface imaging systems can be found elsewhere.^1^ Although trends may soon be changing in favor of eliminating the placement of skin marks, this tool is currently often still utilized in conjunction with laser alignment to tattoos. Radiographic imaging for image guided radiotherapy (IGRT) is still performed to ensure the precision of treatment delivery based on internal anatomy.^2^

IGRT is an essential component of SBRT which employs immobilization devices and image localization techniques to treat small targets using hypofractionated dose regimens and millimeter PTV margins.^3^ In the absence of optical surface imaging, it is common to initially position the patient based on skin marks and lasers, use orthogonal kV images to check overall alignment and match bony anatomy or fiducial markers, and finally refine target localization based on volumetric information from a cone beam CT (CBCT) scan.^4^ To streamline the process, some centers bypass orthogonal imaging before CBCT. While this can be efficient if the initial patient position is adequate, it can also lead to increased patient imaging dose and extended setup time if alignment discrepancies, such as hip rotations or mispositioned extremities, cannot be corrected with automated couch movements and require re‐acquisition of the CBCT to confirm satisfactory alignment prior to treatment. With surface guided radiotherapy (SGRT), positioning can be refined based on real time feedback during initial in‐room setup, providing therapists the capability of detecting and correcting possible rotations or large translational discrepancies before leaving the room to acquire the CBCT. It is clear that SGRT cannot replace internal imaging for SBRT, but quantifying the effects of adding SGRT to the traditional IGRT chain for SBRT (referred to as SG‐SBRT for the remainder of the text) can help elucidate the benefits of this technology. There is literature describing the benefits of utilizing SGRT for deep inspiration breath‐hold treatments of left‐sided breast cancer patients,^5^, ^6^, ^7^, ^8^, ^9^ other breast cancer treatments,^10^, ^11^, ^12^, ^13^, ^14^, ^15^ and stereotactic radiosurgery,^16^, ^17^, ^18^, ^19^ but limited publications on its use for other sites or for initial positioning of SBRT patients.^20^, ^21^ The aim of this retrospective study is to establish the utility of optical surface imaging for initial patient setup in SBRT treatments and to formulate a proposed initial positioning process by studying the impact of orthogonal kV imaging when SG‐SBRT is used and the effects of reference surface type selection (from treatment planning CT versus camera‐acquired in the room) on its performance.

## 2 Methods and Materials

### 2.1 Patient selection and simulation

The use of patient data was reviewed by the Virginia Commonwealth University Institutional Review Board and deemed exempt. This study includes 63 SBRT patients (284 fractions) treated between 2015 and 2016 on a Varian Truebeam (Varian, Palo Alto, California) linear accelerator with a standard (non‐6DOF) table and an AlignRT system (Vision RT Ltd, London, UK, Version 5.0.1747) with standard definition cameras. This system consists of three pods, with two cameras each. Each pod also contains a projector that emits a pseudo‐random speckled pattern of red light. The system uses this pattern to reconstruct the topography of the patient or object in its field of view. The resulting surface is then rigidly aligned to a reference surface based on a user‐defined region of interest (ROI). For a more extensive description of the system, refer to the literature.^1^

Patient data were divided into two cohorts based on whether or not surface imaging guidance was included in their treatment. The non‐SG‐SBRT group, treated in 2015 prior to clinical implementation of AlignRT, includes 37 patients (171 fractions). The SG‐SBRT group consists of 26 patients (113 fractions) treated in 2016. All treatment courses ranged from 3 to 5 fractions, and treatment sites included primary and metastatic cancers of the lung, liver, spine, pancreas, and lymph nodes. Table 1 summarizes the information of the patient treatments included in this study. Some treatments were planned and delivered with the use of an active breathing coordinator (ABC) (Elekta Limited, Crawley, UK). For those patients, the planning CTs were obtained during inspiration breath hold. Patients treated in free breathing were simulated using a 4DCT scan with the 30% phase of the scan used as the primary image set to represent the mid‐ventilation position. Spine patients were simulated in free breathing since respiratory motion does not affect the location of the target. Planning CT scans were acquired with a Philips Big Bore (Philips, Amsterdam, Netherlands) and the techniques used varied between 120 and 140 kVp depending on the patient’s size and treatment site and 280 mAs for standard simulations and 600 mAs for 4DCTs, with 3 mm slice thickness for all scans, except spine (1.5 mm). The patients included in this study were planned in Pinnacle (Philips Radiation Oncology Systems, Fitchburg, Wisconsin, Version 9.6) and all external body contours were automatically created by using an outside‐patient air threshold of 0.6 g/cm^3^. This structure, along with the treatment plan information, was sent to AlignRT using the RTPLAN and RTSTRUCT DICOM files.

**Table 1:** Characteristics of the patient treatment fractions included in each group (SG‐SBRT: SBRT with surface image guidance, non‐SG‐SBRT: traditional SBRT).

	SG‐SBRT	Non‐SG‐SBRT
Total Number of Fractions	113	171
*Breakdown by Anatomical Region*		
Chest	55	112
Abdomen	48	32
Bones	10	27
*Breakdown Based on Breath‐Hold Usage*		
ABC	50	10
No ABC	63	161

SBRT, stereotactic body radiotherapy.

### 2.2 Patient setup workflows

**Figure 1:**
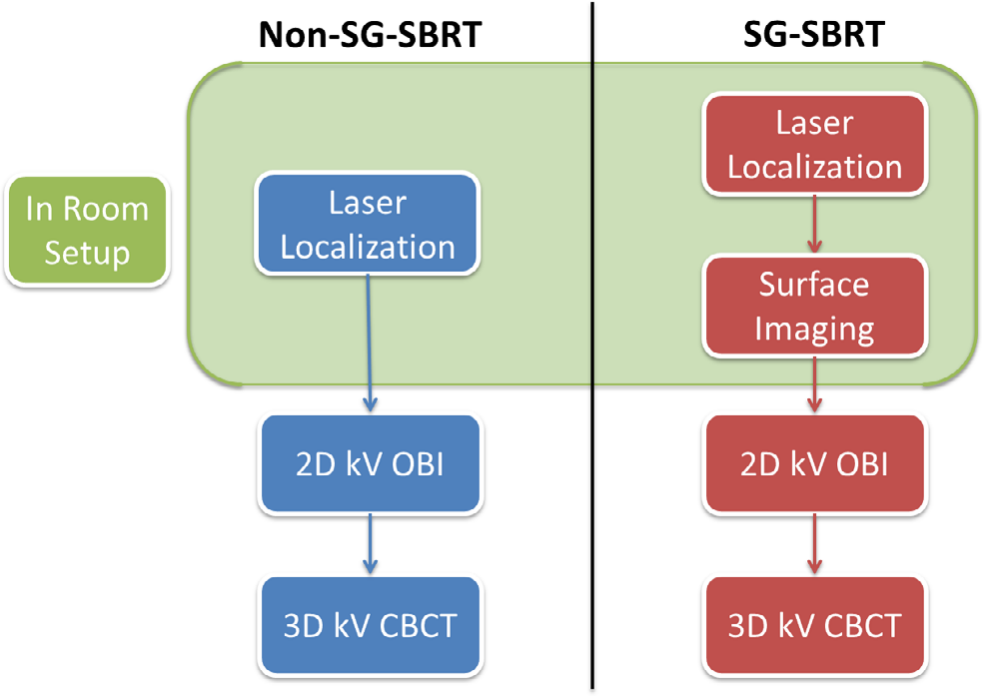
Diagram of SBRT patient in‐room setup and imaging workflow with and without the inclusion of surface imaging guidance (SG). SBRT, stereotactic body radiotherapy.

We investigated the differences between two patient setup workflows: the original procedure (non SG‐SBRT) and the new one (SG‐SBRT). The original clinical workflow involved laser alignment to patient skin marks, with couch shifts to the treatment isocenter if needed, followed by orthogonal kV imaging to correct for translational and rotational setup deviations with respect to bony anatomy. A CBCT was obtained for final target localization prior to the delivery of SBRT. In the new workflow, AlignRT was introduced after laser alignment and before kV imaging to refine the patient’s position in the room (Fig. 1). Although skin mark alignment can be replaced with SG, this step was kept in the new workflow for easier implementation of surface imaging as therapists were still growing accustomed to the system.

**Figure 2:**
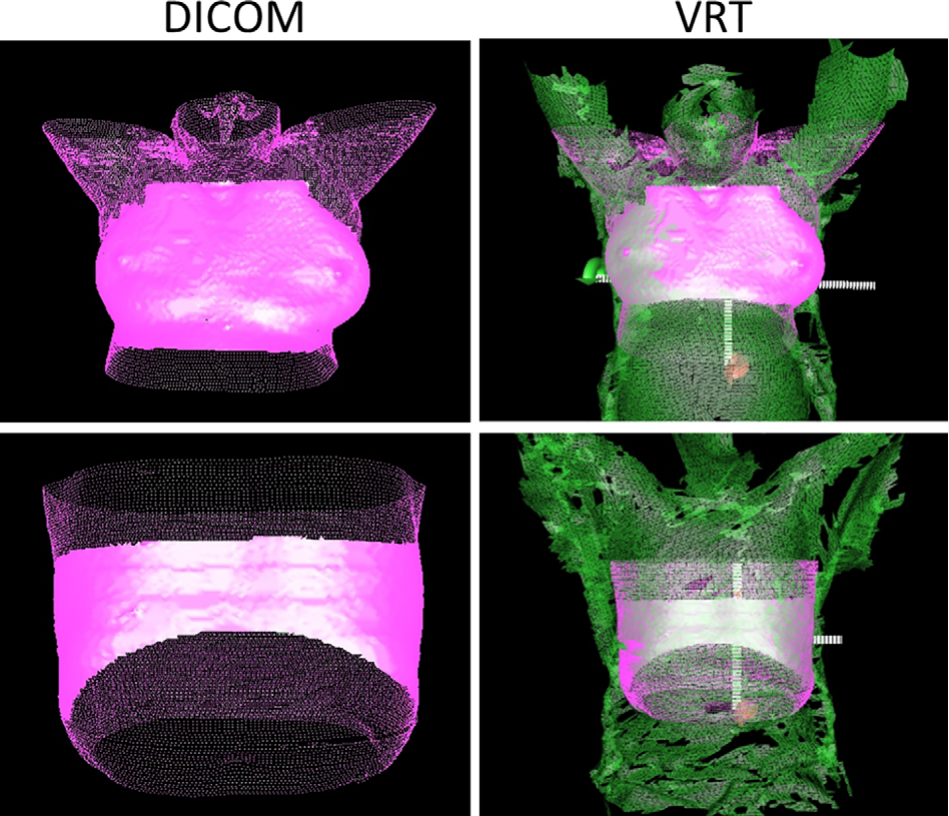
Sample regions of interest (ROIs) drawn on the DICOM reference surfaces (left, pink) and compared to VRT reference surfaces (right, green) in AlignRT.

When using AlignRT for initial setup, an in‐room monitor displays the adjustments needed to correct the patient position in real‐time using a continuous feedback loop. These adjustments are given as three translational (vertical, longitudinal, lateral) and three rotational (yaw, roll, pitch) deltas based on an automatic rigid registration between the real‐time surface of the patient in the room and the selected reference. As mentioned in the introduction, the reference surface can be based on the external body contour of the planning CT (DICOM reference), or acquired using the in‐room optical cameras (VRT reference). The registration only focuses on the area encompassed by the user‐defined region of interest (ROI). Both the accuracy and refresh framerate of the deltas depend on the ROI used for registration. In our workflow, the DICOM reference was always used for initial positioning throughout the treatment, and the ROI was defined following vendor recommendations for different treatment sites (Fig. 2). Prior to clinical use each day, therapists were instructed to perform the vendor‐recommended daily test verification on the surface imaging system to ensure performance was satisfactory (root‐mean‐square position of the isocenter as measured by the system was within 1mm of calibration). If this test showed a deviation beyond the expected value, the system was recalibrated. When positioning patients, therapists were asked to achieve delta values as close to zero as possible before completing the in‐room portion of the setup phase. After refining the patient position with surface imaging, the remaining setup proceeded as usual, with orthogonal kV imaging followed by CBCT. After the treatment position was confirmed based on CBCT, a VRT reference image was captured for intrafraction treatment monitoring.

For either workflow, due to the lack of 6DOF capabilities of the treatment couch, therapists manually adjusted the patient to correct rotational discrepancies deemed large enough to affect the quality of the treatment delivery upon CBCT inspection by the physicist and attending physician. Any time a rotational modification was performed, a second CBCT scan was then acquired to verify the adjusted patient position. Per department policy, an additional CBCT scan is also required to confirm the patient’s alignment prior to treatment if translational shifts on imaging are found to be larger than 8mm in any direction, or 15mm when the absolute value of all three translational shifts are summed. This policy is in place to ensure that the patient’s position is still satisfactory before starting treatment after large imaging shifts have been applied.

### 2.3 Data analysis

The 284 SBRT fractions in this analysis included 171 fractions treated prior to the clinical implementation of surface imaging (original workflow, non‐SG‐SBRT), and 113 treated with the inclusion of AlignRT (SG‐SBRT workflow).

To assess the impact of SG on initial setup, we compared the difference in the initial couch position after in‐room alignment but before kV imaging, to the treatment couch position after final target localization using CBCT, for setups with and without surface imaging. The shifts applied based on every image registration at the treatment machine are automatically saved with each image in the record and verify system, Aria (Varian, Palo Alto, California, Version 11). Hence, these provide the difference in the couch position before and after imaging. The absolute value of these differences was used in the analysis. Although this is a simple analysis, with limitations that will be discussed in a latter section, it is a useful quantitative metric to compare in‐room positioning performance of AlignRT versus laser localization alone. The time difference between the two workflows could not be quantified since the record and verify system has no way to track the time taken to perform laser localization or surface imaging adjustments. The necessity of orthogonal kV imaging in the setup chain for each group was investigated by analyzing if the shifts from orthogonal kV images led to smaller shifts on CBCT. Orthogonal planar kV imaging was deemed to be necessary if its inclusion reduced CBCT shifts to below the threshold defined by the department’s re‐imaging policy described in the previous section. The number of CBCTs required to achieve the final treatment position for each fraction was also recorded. Since 6DOF registration was not available to register the CBCT to the planning CT in real time, this alignment was performed offline with MIM 6.9.2 (Cleveland, OH). An automatic rigid registration was performed for each fraction using a volume‐of‐interest to focus the alignment around the treatment area, if general alignment led to a registration considered unacceptable for treatment. For fractions with multiple CBCT scans, only the first scan was used to calculate the rotational discrepancies to reflect the patient position based on surface imaging guidance prior to any corrections based on volumetric internal imaging. Rotations for each fraction were recorded and analyzed to compare the values between the two cohorts.

Since AlignRT allows users to choose either the DICOM or VRT surfaces as references during setup, the effect of this choice on patient positioning was also investigated. Although our workflow dictated positioning patients to the DICOM reference every time, the daily VRT surfaces acquired for intrafraction monitoring provided the necessary data to perform an offline analysis of the effects of reference surface selection. In order to quantify the differences between the DICOM and the daily VRT surface positions, these two surfaces must be registered to each other to obtain the translational and rotational differences between the two. To measure the interfraction consistency of the daily VRT surfaces acquired throughout the course of treatment, each daily VRT surface must be registered to that of the previous fractions. These off‐line registrations were performed using the “Retrospective analysis” module provided by Vision RT through a research agreement. This module allows the user to load the surfaces captured with the system and compare them to any of the other stored surfaces. It utilizes the same registration algorithm as the clinical AlignRT package and provides the same set of translational and rotational deltas one would obtain in clinical mode.

**Figure 3:**
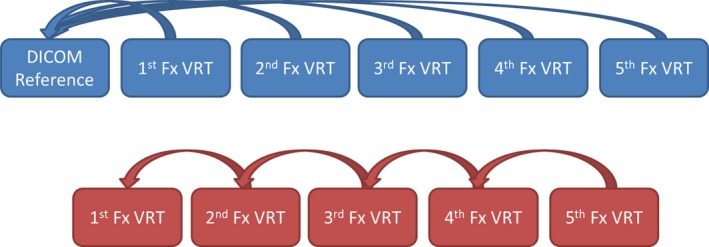
Comparison of differences in daily VRT reference captures compared to 1) the DICOM reference surface (top) and 2) the VRT reference capture from the previously treated fraction.

The differences in registration between the VRT surfaces captured at each fraction were evaluated to assess day‐to‐day surface reproducibility, both against the DICOM reference surface, as well as to the VRT surface captured at each previously treated fraction (Fig. 3). While the first comparison of each daily VRT reference to the DICOM surface reflects the actual deviations of daily surfaces to the ideal patient position from the treatment plan, the second comparison was performed to evaluate if updating the reference surface at every fraction could improve setup. The results from this analysis can be used to make a better decision on what reference surface to use when implementing SG‐SBRT.

The p‐values of the differences between the two data sets for all parameters studied were calculated using a Wilcoxon Rank‐Sum test (α = 0.05) since the data are not normally distributed.

## 3 Results

**Figure 4:**
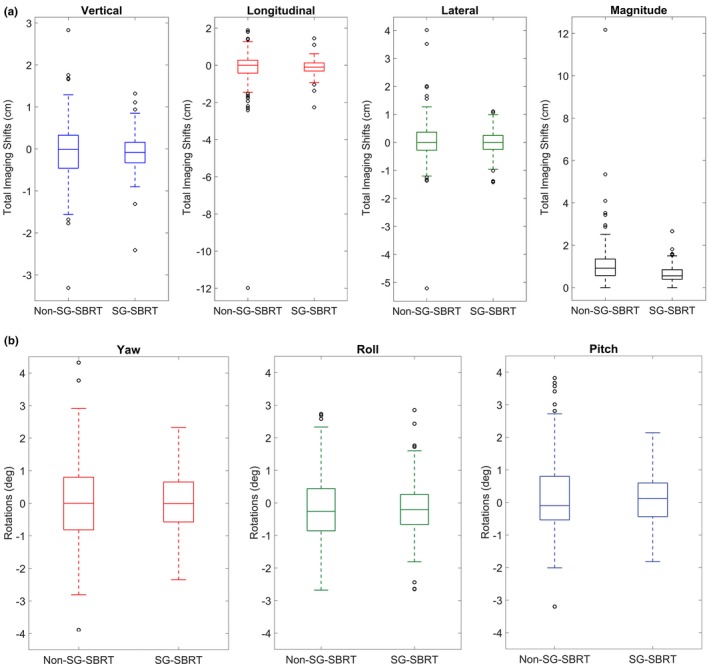
(a) Box plots demonstrating the differences between initial (pre‐radiographic imaging) and treatment couch positions for each translational direction and vector magnitude, for non‐SG‐SBRT and SG‐SBRT. (b). Box plots displaying the residual rotations for patient setups of non‐SG‐SBRT and SG‐SBRT. SBRT, stereotactic body radiotherapy.

A comparison of the initial couch position at the start of kV imaging to final couch position after CBCT imaging demonstrates a smaller range and median deviation in all three translational directions and vector magnitude when optical surface imaging is included in the workflow. Table 2 displays the absolute median, quartile 1, quartile 3, and maximum couch position differences for the absolute value of all individual translations and magnitudes of the two cohorts. The minimum is not shown as it is 0 in all cases. Figure 4(a) presents these data, with their original sign – no absolute value, in box plots, demonstrating the couch position differences for each translation direction and their magnitude, with and without SG. The differences along all translations and magnitude between the two groups are statistically significant. Additionally, the maximum observed deviations in the SG‐SBRT group were much smaller than in the non‐SG‐SBRT group (vertical, longitudinal, and lateral directions were 2.41 cm, 2.26 cm, and 1.42 cm, and 3.31 cm, 11.98 cm, and 5.21 cm, respectively). In total, there were 19 fractions (16.8%) in the SG‐SBRT group and 78 fractions (45.6%) in the non‐SG‐SBRT group where the translational deviation was greater than 1cm. There was also a statistically significant difference in the rotations found amongst the two groups. Table 2 also shows the median, quartile 1, quartile 3, and maximum rotations along the three directions (pitch, yaw, roll). Overall, the rotations for the SG‐SBRT group were smaller in a statistically significant manner, although the maximum roll value calculated for that group was slightly larger than that of the non‐SG‐SBRT arm. Figure 4(b) shows the box plots of the rotations.

**Table 2:** Couch position differences from pre‐orthogonal kV imaging to post‐CBCT localization, for the non‐SG‐SBRT and SG‐SBRT groups.

	Absolute Imaging Couch Position Differences (cm)				Absolute Rotation Differences (deg)			Average number of CBCTs
	Vertical	Longitudinal	Lateral	Magnitude	Pitch	Yaw	Roll	
Non‐SG‐SBRT								
Median	0.41	0.39	0.32	0.92	0.6	0.8	0.7	1.13
Quartile1	0.17	0.14	0.14	0.57	0.32	0.31	0.34	
Quartile3	0.64	0.83	0.67	1.35	1.17	1.32	1.24	
Max	3.31	11.98	5.21	12.17	3.82	4.32	2.73	
SG‐SBRT								
Median	0.26	0.23	0.25	0.56	0.54	0.63	0.54	1.12
Quartile1	0.10	0.11	0.11	0.39	0.27	0.28	0.23	
Quartile3	0.44	0.46	0.41	0.85	0.93	1.09	1.09	
Max	2.41	2.26	1.42	2.66	2.14	2.35	2.85	
*P*‐value, α = 0.05	0.0004*	0.0017*	0.0242*	<0.0001*	0.0404*	0.0270*	0.0336*	0.3115

CBCT, cone beam CT; SBRT, stereotactic body radiotherapy.

The rotational differences shown were calculated off‐line using rigid registration through MIM 6.9.2, for the non‐SG‐SBRT and SG‐SBRT groups. Minimum values not shown since they are all 0.

* denotes statistical significance.

Internal institutional policy dictates that reimaging with CBCT is necessary to confirm the patient’s position prior to treatment if the shifts on the first CBCT are greater than 15mm (absolute value of all translational shifts added together), or larger than 8mm in any one translational direction. Therefore, if a shift of 6 mm was recorded on the orthogonal kVs along the vertical direction for example, and the subsequent shift along that same direction on the CBCT was only 3 mm, the inclusion of planar kV imaging was deemed useful as it kept the vertical CBCT shift below the 8 mm threshold. Based on these guidelines, the addition of planar kV imaging helped in 87 (50.9%) of the non‐SG‐SBRT fractions, and in 21 (18.6%) of SG‐SBRT ones. When investigating if there was a general anatomical area of soft tissue targets (chest or abdomen) that benefited more from the addition of orthogonal kV imaging, the results did not show a difference (see Table 3). Not surprisingly, the addition of orthogonal kV imaging is beneficial for bony targets in the non‐SG‐SBRT group. The difference in the average number of CBCTs between the two groups is not statistically significant.

**Table 3:** Number of fractions, with corresponding percentage in parenthesis, in which the inclusion of orthogonal kV imaging resulted in CBCT shifts below the reimaging thresholds set by the in‐house clinical protocol.

	Number of fractions necessitating the inclusion of orthogonal kV	
	SG‐SBRT	Non‐SG‐SBRT
Total Number of Fractions	21/113 (18.6%)	87/171 (50.9%)
*Breakdown by Anatomical Region:*		
Chest	10/55 (18.2%)	53/112 (47.3%)
Abdomen	10/48 (20.8%)	15/32 (46.9%)
Bones	1/10 (10%)	19/27 (70.4%)
*Breakdown Based on Breath‐Hold Usage:*		
ABC	14/50 (28%)	4/10 (40%)
No ABC	7/63 (11.1%)	83/161 (51.6%)

ABC, active breathing coordinator; CBCT, cone beam CT; SBRT, stereotactic body radiotherapy.

Of the 113 patients in the SG‐SBRT group, 102 had VRT reference captures. Table 4 displays the absolute median, quartile 1, quartile 3, minimum, and maximum differences for the DICOM versus daily VRT references as well as the daily VRT versus previous fraction VRT references for all translations, magnitudes, and rotations. Table 4 also displays the *P*‐values for the two groups, once again calculated with a Rank‐Sum test using an alpha of 0.05. None of the values were found to be statistically significant. In both groups, the largest deviations were in longitudinal translations and pitch rotations.

**Table 4:** Differences in daily camera‐acquired reference surfaces compared to the DICOM references, as well as to the camera‐acquired surface acquired at the previously treated fraction.

Deviations in Reference Surface Comparisons							
	Vertical (cm)	Longitudinal (cm)	Lateral (cm)	Magnitude (cm)	Yaw (°)	Roll (°)	Pitch (°)
DICOM to VRT Reference							
Median	0.20	0.25	0.18	0.46	0.90	0.80	0.90
Quartile1	0.11	0.12	0.08	0.30	0.40	0.30	0.40
Quartile 3	0.36	0.46	0.28	0.71	1.48	1.20	1.70
Min	0.00	0.00	0.00	0.09	0.00	0.00	0.10
Max	0.93	1.19	0.72	1.36	3.20	3.80	6.90
VRT to VRT Reference							
Median	0.18	0.20	0.18	0.44	0.70	0.70	0.80
Quartile1	0.08	0.09	0.09	0.29	0.23	0.40	0.40
Quartile 3	0.32	0.36	0.30	0.66	1.20	1.40	1.58
Min	0.00	0.00	0.00	0.03	0.00	0.00	0.10
Max	0.93	1.12	0.94	1.36	3.10	4.10	7.40
*P*‐value, α = 0.05	0.54	0.10	0.82	0.09	0.73	0.30	0.27

SBRT, stereotactic body radiotherapy.

## 4 Discussion

The results of this retrospective study show that the addition of optical surface imaging into the clinical workflow for SBRT reduces the magnitude of setup deviations between in‐room setup and final CBCT localization. Since all the shifts and rotations are statistically significantly less when using SG‐SBRT than those for laser localization alone, it may be reasonable to use surface imaging as a replacement for laser alignment. The superior accuracy of surface imaging guidance over laser localization has also been demonstrated by Stanley et al. for the magnitude of 3D shift vectors in a study containing 6000 fractions over a large range of treatment sites using the C‐RAD CatalystHD system (C‐RAD, Uppsala, Sweden).^22^ However, publications on the use of surface imaging for initial positioning of SBRT alone remain limited.

The statistically significant differences in shifts (both translations and rotations) indicate potential drawbacks of skin mark and laser localization for initial setup. For instance, for patients with loose skin, these marks can be easily and superficially manipulated to align with the lasers without producing the necessary correction of the patient’s internal anatomy. Because surface imaging systems evaluate several thousands of points on the patient over a region of interest, they provide a more comprehensive and robust method of assessing the patient’s position relative to the plan. Since some surface imaging commercial systems require users to manually select the region of interest, the quality of the setup achieved based on surface imaging will depend on appropriate ROI definition and user proficiency. Nevertheless, the data also demonstrates that surface imaging helps detect large setup errors prior to imaging (see Table 2). For the non‐SG‐SBRT group, 2.9% of fractions had translational differences greater than 3 cm, 5.8% had greater than 2 cm, and 45.6% greater than 1 cm. The SG‐SBRT group did not have any fractions with discrepancies larger than 3 cm, had 1.8% with differences greater than 2 cm, and 16.8% of fractions with differences exceeding 1 cm. Large deviations may arise from patients being set up to incorrect skin marks (from previous treatments, for example), or by inaccurately applying shifts to move from marked to treatment isocenter. These errors become evident with surface imaging and can therefore be avoided.

Our analysis on how SG affects the usefulness of orthogonal kV imaging during treatment setup shows that the contribution from orthogonal kVs decreases with the addition of surface imaging. However, the use of kV imaging still proved necessary to avoid re‐CBCT imaging in almost 19% of the SG‐SBRT fractions, versus almost 51% in the non SG‐SBRT group. This indicates that orthogonal kV imaging could have been excluded from over 80% of the SG‐SBRT fractions studied in this work without impacting the quality of the setup. The sample studied is not large enough to reliably extract characteristics to identify ahead of time what patients will need orthogonal kV imaging. Patients with lesions that correlate more closely to bony anatomy or patients that are larger and have more posterior lesions should generally benefit from orthogonal kV imaging; however, this is not always the case. Because of this, the new workflow established in our clinic indicates that patients should be imaged with orthogonal kV prior to CBCT for the first fraction, and the physicist can then identify if this step adds value or can be bypassed for subsequent treatments. In cases where the use of kV imaging is beneficial, the addition of surface imaging to the process still improves the safety of treatment as it ensures that gross initial positioning errors are avoided, allows for real time intrafraction monitoring during treatment, and highlights topographical anatomical changes that could potentially affect the dose delivery (i.e. swelling, abdominal distention, etc.) as these alter how the surface correlates with the internal structures.^20^ Hence, all SBRT patients in our clinic are monitored with SG, unless there is a factor that impedes it (patient is being treated with a mask, patient does not want to be uncovered during treatment, etc.). An additional benefit of implementing SG‐SBRT for every patient is the possibility of terminating the use of skin marks. Tattoo‐less radiotherapy potentially reduces patient discomfort and stress in a difficult period of their life.^23^ This approach has been heavily discussed among SGRT users at various professional conferences, but very few publications exist in the literature. However, there is published work showing that breast patients have improved body image scores at 1 and 6 months‐post therapy when UV tattoos (invisible in ambient lighting) are used versus conventional dark ink skin marks.^24^ This indicates that bypassing tattoos as part of the setup workflow can potentially improve patient experience.

It is important to discuss the limitations that stem from evaluating the quality of the initial positioning of the SG‐SBRT and non‐SG‐SBRT groups based on the imaging shifts in the record and verify system. Many imaging corrections must be performed manually by the therapists in the room that may shift the patient’s position relative to the position of the couch – e.g. correcting a misplaced extremity, or a manual rotation adjustment. As our method uses the magnitude of differences in kV imaging couch positions between the first image and last CBCT, these in‐room manual corrections would introduce alterations from the initial position that would not be reflected on the couch coordinates. These uncertainties are unavoidable in the design of the study due to its retrospective nature. With the study being retrospective, it is also impossible to acquire time estimates of how long therapists spent during initial setup performing laser localization with skin marks alone versus SG prior to imaging. This information would also be valuable in comparing the two processes, since if one is considerably longer than the other, assuming the comfort level of the users is the same for both, this should be factored into the evaluation as it can affect clinical workflow and patient comfort.

While the reference surface study found that the mean deviations between the DICOM surface and VRT captures were small, the maximum deviations were near the order of 1.0 cm in translations and >3.0° for rotations in both groups. These deviations may be the result of varying causes including periodic intrafraction motion such as free breathing during setup and reference capture, transient anatomical changes such as bloating, and different proficiency levels of the therapists in the use and interpretation of the surface imaging feedback. Since the data included in this study encompasses the early use of surface imaging in our clinic, some of the reference captures acquired with the cameras have portions of missing anatomy due to gantry occlusion. Surfaces were inspected prior to inclusion in this study, and only surfaces where the anatomy encompassed by the ROI was complete were used. Partly incomplete anatomy might affect registration accuracy, but its effects on these results should be minimal as surfaces with significant occlusion were discarded.

**Figure 5:**
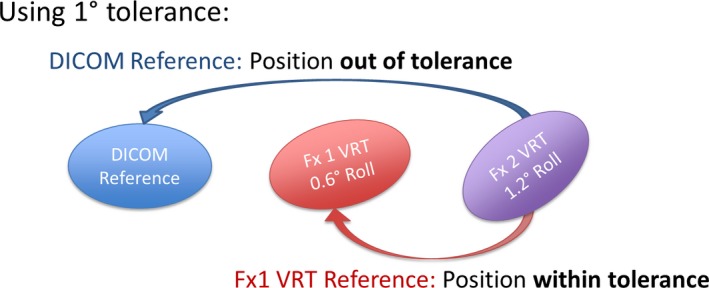
Illustration of the potential propagation of systematic errors when performing initial setup for a patient based on a surface imaging system acquired daily reference surface.

Longitudinal translations and pitch in the reference surface study demonstrated maximum differences greater than 1.0 cm and 6.0° respectively. The drastic deviation in pitch may in part be caused by the difficulty in performing corrections in that rotational axis without the use of a 6DOF couch. Patient nonconformity and varying angles of the pelvis or lumbar spine due to different muscle contraction from changing levels of comfort and stress between simulation and treatment are all compounding factors that can contribute to nonreproducibility. Further differences can be introduced by the use of breath hold for patients that are not coached properly or have difficulty undergoing the process in a reproducible manner. Even though no statistically significant difference was found in our study between initial positioning to DICOM versus VRT surfaces, it is important to understand that subsequent setup to the VRT reference rather than the DICOM reference may systematically propagate any residual deviations from the planned position captured at the previous fraction. This is demonstrated in Fig. 5, which shows a hypothetical situation in which the VRT reference capture at fraction 1 is obtained, after internal imaging verification, when a 0.6° residual roll is present. At fraction 2, the patient setup includes a 1.2° roll in the same direction with respect to DICOM. Assuming a 1.0° tolerance for rotations, the setup would be displayed correctly as being out of tolerance if the DICOM reference is used. However, relative to the VRT reference at fraction 1, the deviation would be calculated as 0.6°. Although the initial 0.6° rotation might have been acceptable for treatment, the increased 1.2° could differ too much from the desired treatment position, thus leading to a rotational discrepancy that would require the acquisition of a second CBCT after its correction.

On the other hand, the DICOM surface is also susceptible to certain uncertainties and may not be a perfect representation of the ground truth. In our clinical protocol, all SBRT CT simulation scans for nonspine SBRT are acquired using a 3.0 mm slice thickness. Thus, the resolution in the cranio‐caudal direction includes more coarse interpolation in the generation of the DICOM surface compared to the axial plane and to the finer resolution of the AlignRT cameras used to capture the VRT surface. This could be a contributing factor in the larger deviations observed along the longitudinal direction when comparing the DICOM and VRT surfaces. While breathing motion artifacts at simulation may introduce uncertainties in the segmentation of the external contour for patients being treated in the thoracic and abdominal regions, all lung, liver, and pancreas SBRT patients were simulated either with a 4DCT or active breathing coordinator. Only 17 fractions over two T‐spine, one adrenal gland, and one para‐aortic node patient were simulated without accounting for breathing motion. The choice of patient air threshold in the treatment planning system also has implications on the segmentation of the surface, although this effect has been shown to be submillimeter in other studies.^25^ When available, these differences could be evaluated by obtaining a reference capture using surface imaging cameras installed in the simulation room and comparing the agreement between the two surfaces.

As this study evaluates the setup accuracy for SBRT treatments, our data focuses on hypofractionated regimens, so these conclusions may not hold true for more conventionally fractionated courses where the patient may demonstrate physical changes over time, such as tumor shrinkage or weight loss, that may cause considerable deviations from the DICOM surface generated from the initial simulation scan. When such deviations are not large enough to call for resimulation and adaptive replanning, a VRT reference captured prior to treatment would allow for more accurate daily setup as it reflects these external changes, but is acquired after internal anatomical verification. Otherwise, the VRT reference should primarily serve as a tool for intrafraction monitoring. When the treatment position does not perfectly replicate the DICOM reference due to the aforementioned interfraction variations, any residual deviations after localization with volumetric internal imaging may mask small changes in the patient’s position. If the DICOM reference is used for intrafraction monitoring, patient motion during treatment may go unnoticed.

As this is an offline retrospective analysis, the surface study is a simplified representation of the potential differences between the DICOM and VRT reference surfaces. It does not include data from actual patient setup to daily reference captures, so it lacks the variations that the manual alignment would introduce when therapists process the feedback from the system in real time as they are adjusting the patient’s position. To truly assess whether such an effect exists, a prospective trial randomizing patients to daily setup after the first fraction using the DICOM versus the previously acquired VRT reference surface must be conducted to determine whether there is a statistically significant difference in shifts between the two methods.

Lastly, the data collected from the treatments delivered with AlignRT began when the system was newly implemented and thus, presents a gradual refinement in procedural definition and proficiency of therapists in its use. While familiarity with the system gradually improved over time, AlignRT was installed on a single TrueBeam linear accelerator at our institution. As a result, proficiency in setup with the system was not uniform among radiation therapists, who routinely rotate assignments between treatment machines. Thus, the patient setups with AlignRT may not demonstrate maximum proficiency attained in comparison to setup with laser localization, which is a standardized skillset among all radiation therapists at our institution. Despite this, the SG‐SBRT cohort still shows a statistically significant decrease in positional discrepancies when compared to the non‐SG‐SBRT group.

## 5 Conclusions

The addition of surface imaging was found to improve the precision and safety of initial patient setup for SBRT treatments. Patients in the SG‐SBRT cohort had translations and rotations between the initial position and treatment position that were statistically significantly less than the non‐SG‐SBRT cohort. Although the number of CBCTs acquired for setup was similar in both groups, the addition of orthogonal kV imaging to the initial setup process was only valuable to keep the CBCT shifts below re‐imaging thresholds for less than 19% of the SG‐SBRT fractions compared to 51% of the non‐SG‐SBRT ones. Hence, the inclusion of orthogonal kV imaging as part of the initial setup process could be re‐evaluated for SBRT patients when using surface image guidance. The choice of reference surface for initial positioning with surface imaging does not make a statistically significant difference in the outcome; however care should be taken to avoid systematic propagation of positional discrepancies when using a camera‐acquired instead of a DICOM reference.

## Conflict of Interest

Dr. Padilla reports nonfinancial support from Vision RT, during the conduct of the study; nonfinancial support from Vision RT and a grant from Philips, outside the submitted work. Mr. Leong has nothing to disclose.
